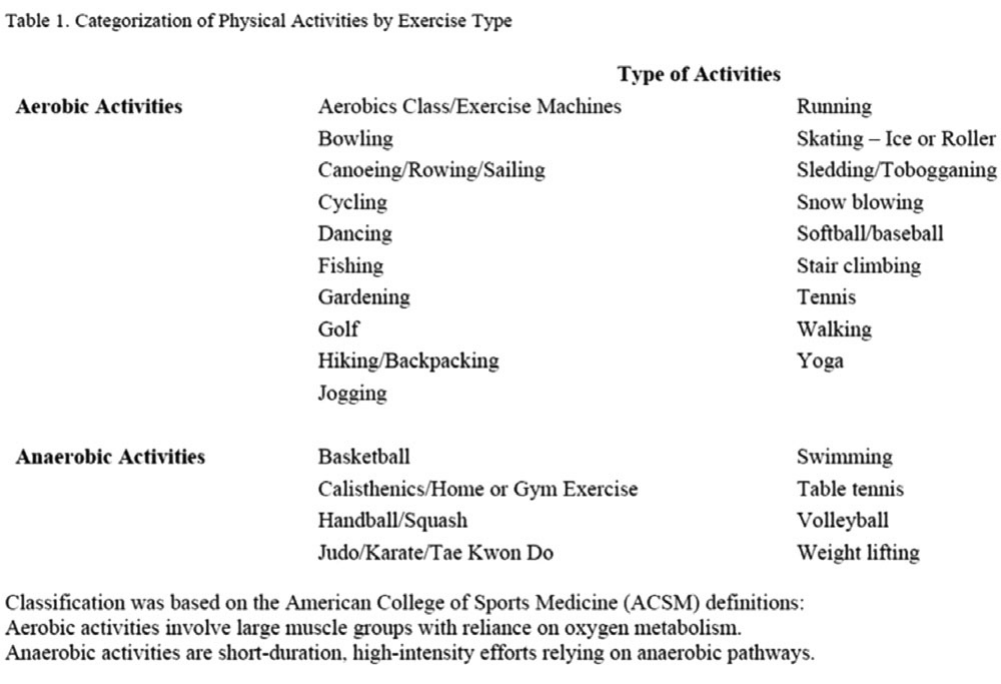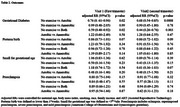# Oral Concurrent Session 2 – Diabetes: Wednesday, February 11, 2026 1:00 PM – 3:30 PM

**DOI:** 10.1002/pmf2.70147

**Published:** 2026-02-09

**1:00 PM – 1:15 PM**

## 19 | Predictive Ability of Early Pregnancy Glycemic Markers for Gestational Diabetes and Large for Gestational Age

Camille E. Powe^1^; Margaret Banker^2^; Patrick M. Catalano^3^; Francesca Facco^4^; William A. Grobman^5^; Erin S. LeBlanc^6^; William L. Lowe^7^; Audrey Merriam^8^; Mirella Mourad^9^; Caryn E.S. Oshiro^10^; Uma M. Reddy^11^; Dwight J. Rouse^12^; Jennifer Sherr^13^; Alexandra C. Spadola^14^; Kimberly K. Vesco^6^; Erika F. Werner^14^; Lynn M. Yee^15^; Noelia Zork^11^; Denise Scholtens^15^; Maisa Feghali^16^

^1^Massachusetts General Hospital, Boston, MA; ^2^Northwestern Feinberg School of Medicine, Chicago, IL; ^3^MGH REU, Boston, MA; ^4^UPMC, UPMC, PA; ^5^Warren Alpert Medical School of Brown University, Providence, RI; ^6^Kaiser Permanente Center for Health Research, Portland, OR; ^7^Northwestern University, Northwestern University, IL; ^8^Yale School of Medicine, Yale School of Medicine, CT; ^9^Division of Maternal‐Fetal Medicine, Department of Obstetrics and Gynecology, Columbia University Irving Medical Center, New York, NY; ^10^Kaiser Permanente Hawaii, Center for Integrated Health Care Research, Honolulu, HI; ^11^Columbia University Irving Medical Center, New York, NY; ^12^Women & Infants Hospital of Rhode Island / Warren Alpert Medical School of Brown University, Providence, RI; ^13^Yale School of Medicine, New Haven, CT; ^14^Tufts University School of Medicine, Boston, MA; ^15^Northwestern University Feinberg School of Medicine, Chicago, IL; ^16^Magee Women's Research Institute, University of Pittsburgh, Pittsburgh, PA

**Objective**: To evaluate hemoglobin A1c (A1c) and other shorter‐term glycemic markers as predictors of gestational diabetes (GDM) and large for gestational age birthweight (LGA).

**Study Design**: In a prespecified secondary analysis of a prospective cohort study at 9 US centers, we measured A1c, glycated albumin, and 1,5‐anhydroglucitol at 10–14 weeks’ gestation in gravidas with singleton pregnancies and no pre‐existing diabetes. Outcomes were GDM at 24–28 weeks’ gestation (Carpenter‐Coustan criteria) and LGA (birthweight > 90%). We developed predictive models using glycemic markers and clinical factors (age, BMI, and history of GDM or macrosomia) in training data from 4 centers, with validation in data from the remaining 5 centers. We selected optimal thresholds for glycemic markers in combination with clinical factors by maximizing area under the ROC curve in logistic regression models. We then calculated the positive predictive value (PPV) at a pre‐specified negative predictive value (NPV) threshold and prevalence (8.3% for GDM, 10% for LGA, based on US national data) to assess clinical utility of models for identifying at‐risk individuals in early pregnancy. We compared PPVs from models with glycemic markers and clinical factors to those from models with clinical factors alone.

**Results**: Among 2178 participants, 15.4% were diagnosed with GDM and 11.3% had LGA infants. No glycemic marker improved prediction of GDM or LGA compared to clinical factors alone (Figure). For prediction of GDM, PPV for the clinical model was 12.2% at NPV of 96% and 8.3% prevalence. The PPVs at the same NPV for glycemic markers ranged from 12.5–14.5% (Table). For prediction of LGA, PPV for the clinical model was 12.4% at NPV of 95% and 10% prevalence. The PPVs at the same NPVs for glycemic markers ranged from 13.2–16.7% (Table). In validation, there was no significant difference between PPVs of models with glycemic markers versus those with clinical factors alone.

**Conclusion**: Neither HbA1c nor other shorter‐term glycemic markers at 10–14 weeks’ gestation improved prediction of GDM or LGA beyond clinical factors alone.

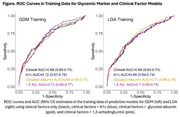

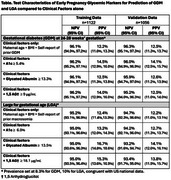

**1:15 PM – 1:30 PM**

## 20 | A RCT of Patient‐Led Insulin Titration for Glycemic Management With Gestational Diabetes Mellitus

Xiao‐Yu Wang^1^; William A. Grobman^2^; Jiqiang Wu^1^; Rita Suzawa^1^; Jenna Kralik^1^; Taryn L. Summerfield^1^; Shaylyn Vickers^1^; Brenda Widmayer^1^; Melissa Rainier^1^; Julie Somppi^1^; Lisa Buccilla^1^; Bridget Iadicicco^1^; Elizabeth O. Buschur^1^; Steven Gabbe^1^; Mark B. Landon^3^; Kartik K. Venkatesh^3^

^1^The Ohio State University Wexner Medical Center, Columbus, OH; ^2^Warren Alpert Medical School of Brown University, Providence, RI; ^3^The Ohio State University Wexner Medical Center, The Ohio State University Wexner Medical Center, OH

**Objective**: We conducted a RCT to compare whether patient‐led insulin titration (intervention) compared with provider‐led insulin titration (control) resulted in improved glycemic management and pregnancy outcomes for individuals with GDM.

**Study Design**: EMPOWER was a single‐center, non‐blinded RCT among individuals with GDM requiring insulin between 20–32 weeks. Intervention participants self‐titrated long‐acting insulin starting at 10 units nightly and increased or decreased by 2 units per fasting glucose above or below 70–95 mg/dL, respectively, every day. Control participants started insulin with weekly titration at the provider's discretion. The primary outcome was mean fasting glucose in the 36^th^ week or the week prior to delivery for preterm deliveries. Secondary outcomes included pregnancy outcomes and patient‐reported measures. With 80% power, 56 individuals needed to be randomized to demonstrate at least a 15% difference in mean glucose.

**Results**: Of 89 screened and eligible individuals, 56 were randomized (29 intervention/27 control). The median duration from starting insulin to delivery was 7.7 weeks. Patient‐led insulin titration resulted in a similar weekly average glucose before delivery compared with provider‐led titration (88.8 vs. 90.3 mg/dL; β coef.: −1.50 mg/dL; 95% CI: −5.50–2.50), but was associated with lower odds of LGA (6.5 vs. 39.3%; OR: 0.10, 95% CI 0.02–0.55) and NICU admission (9.7 vs. 32.0%; OR: 0.23, 95% CI: 0.05–0.98) (Table). Patient‐led insulin titration resulted in more rapid normalization of fasting glucose than provider‐led insulin titration (mean: 1.8 vs. 2.5 weeks; HR: 1.48, 95% CI: 1.16–1.90) (Figure). Other pregnancy and patient‐reported outcomes did not differ between the groups.

**Conclusion**: Patient‐led self‐insulin titration for GDM did not result in lower fasting glucose before delivery compared with provider‐led insulin titration but was associated with a significantly lower risk of LGA and NICU admission and a quicker time to achieving glycemic control. These data support the need for patient‐centered GDM treatment strategies.

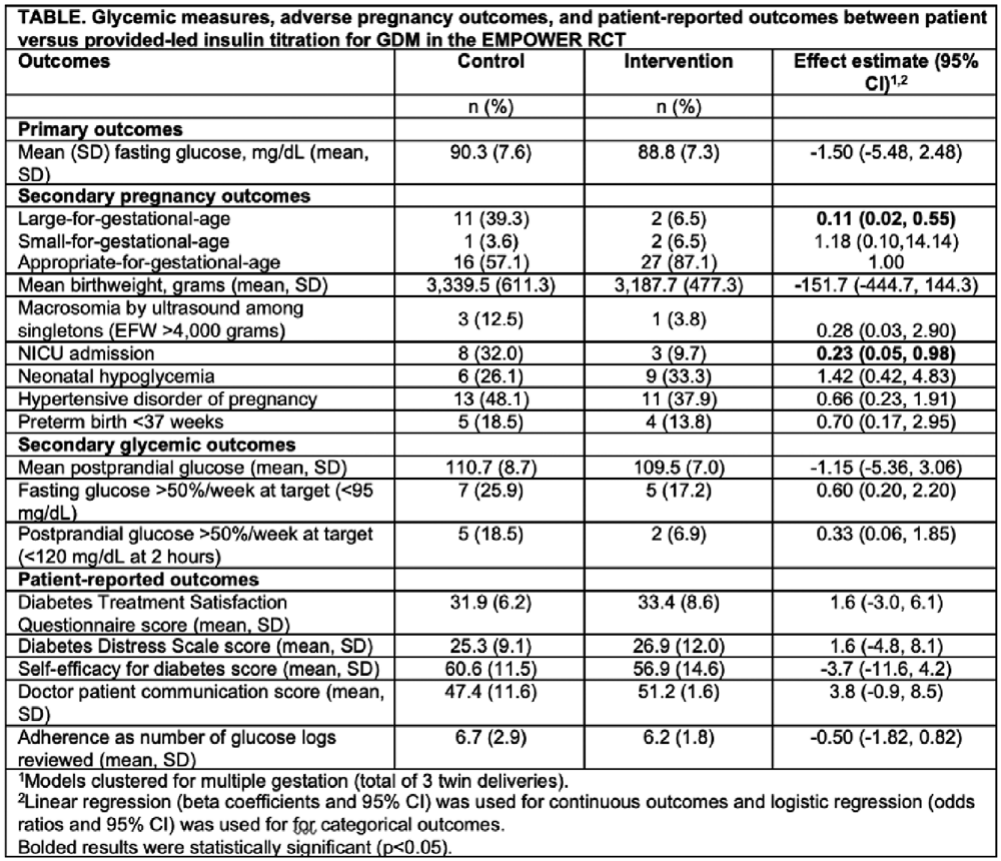

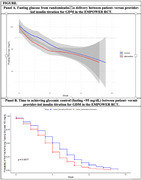

**1:30 PM – 1:45 PM**

## 21 | **Disposition Index During Pregnancy as a Predictor of Insulin Requirement in Women With Gestational Diabetes**

Sohei Hamazaki; Hiroshi Yamashita; Ichiro Yasuhi

NHO Nagasaki Medical Center, Omura, Nagasaki

**Objective**: The disposition index (DI) quantifies pancreatic β‐cell compensation for insulin resistance and is a robust predictor of glucose intolerance and type 2 diabetes outside of pregnancy. However, its clinical significance in gestational diabetes (GDM) remains unclear. This study aimed to evaluate whether a lower DI at the time of GDM diagnosis is associated with the need for insulin therapy during pregnancy.

**Study Design**: We conducted a prospective cohort study of singleton GDM pregnancies managed at a Japanese tertiary perinatal center. GDM was diagnosed by the IADPSG criteria. At the time of diagnostic 75g oral glucose tolerance test (OGTT), plasma insulin and glucose levels were measured. DI was calculated using OGTT values. Patients were stratified into DI quartiles, and the association between DI and insulin requirement was assessed using logistic regression, adjusting for maternal age, prepregnancy body mass index (BMI), family history of diabetes, gestational age at OGTT, glucose levels, and HbA1c.

**Results**: Of 247 participants (mean age 34 years, mean BMI 23.5 kg/m^2^, 48% with family history), 51% required insulin therapy. Women who required insulin had significantly lower DI than those managed with diet alone (1.49 ± 0.41 vs. 1.84 ± 0.58, *p* < .0001). The area under the ROC curve for DI predicting insulin use was 0.73. Insulin requirement increased across decreasing DI quartiles (Figure). Women in the lowest quartile (Q1) had a significantly higher adjusted odds of insulin therapy compared to higher quartiles (aOR 5.79, 95% CI 1.53–22.0) (Figure).

**Conclusion**: Lower DI at GDM diagnosis is a strong independent predictor of insulin requirement during pregnancy. These findings suggest that impaired β‐cell compensation is a key pathophysiologic trait among women needing insulin and may represent a high‐risk subgroup for postpartum diabetes.

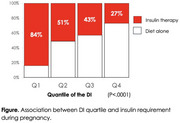

**1:45 PM – 2:00 PM**

## 22 | **Are Hofbauer Cells Inflammatory in Placentas From Pregnancies Complicated by Gestational Diabetes Mellitus?**

Morgan E. Wasickanin^1^; Rachel Watkin^2^; Joshua Monson^2^; Sarah Ligon^2^; Samantha Ocampo^3^; Hillary Kinsman^2^; Jennifer Damicis^2^; Katherine Free^2^; Lydia Bettridge^2^; Emily D. Sheikh^2^; Robert Walton^2^; Peter Napolitano^4^; Nicholas Ieronimakis^2^

^1^Madigan Army Medical Center, Fort Bragg, NC; ^2^Madigan Army Medical Center, Tacoma, WA; ^3^Oregon Health & Science University, Portland, OR; ^4^University of Washington, Seattle, WA

**Objective**: The placental immune system is critical for fetal development and maternal health. Placental immune responses are mediated by resident macrophages, Hofbauer cells (HBCs). Perinatal complications are observed in Gestational Diabetes (GDM), even in the setting of optimal glycemic control. Placental inflammation may underlie these complications and vary between GDM disease classifications (A1GDM vs. A2GDM). It is unclear whether HBCs or maternal leukocytes mediate placental inflammation in the setting of GDM. We hypothesized that HBCs are responsible for placental inflammation and aimed to examine whether this cell population is altered in GDM.

**Study Design**: Placentas from term, unlabored, cesarean births were sectioned from non‐diabetic, A1GDM, and A2GDM pregnancies (*n* = 9‐11/condition). Immunostaining was performed for antigens corresponding to HBCs (CD163) and maternal leukocytes: neutrophils (CD15) and lymphocytes (CD56). Each cell type was counted within 1.2 mm^2^ regions of intervillous and normalized to the tissue area, excluding vascular spaces. HBC inflammatory status was determined by co‐staining CD163 with pro‐inflammatory (CD86) and anti‐inflammatory (CD206) markers. The percentage of HBCs co‐labeled with CD86 or CD206 was quantified using confocal microscopy.

**Results**: The number of HBCs was significantly greater in A1GDM and A2GDM compared to non‐diabetic samples (Figure 1). The number of maternal leukocytes was low (<10 cells per region) and did not change. The proportion of pro‐inflammatory HBCs significantly increased in both GDM classifications, whereas anti‐inflammatory HBCs did not change (Figure 2).

**Conclusion**: We observed a substantial increase in the number of HBCs and their pro‐inflammatory status with GDM. There was no difference in cells identified by markers associated with maternal leukocytes. The elevation of pro‐inflammatory HBCs may promote placental injury and dysfunction. Further research is needed to evaluate the impact of pro‐inflammatory HBCs on adverse outcomes in GDM and the potential role for anti‐inflammatory treatment modalities.

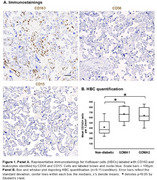

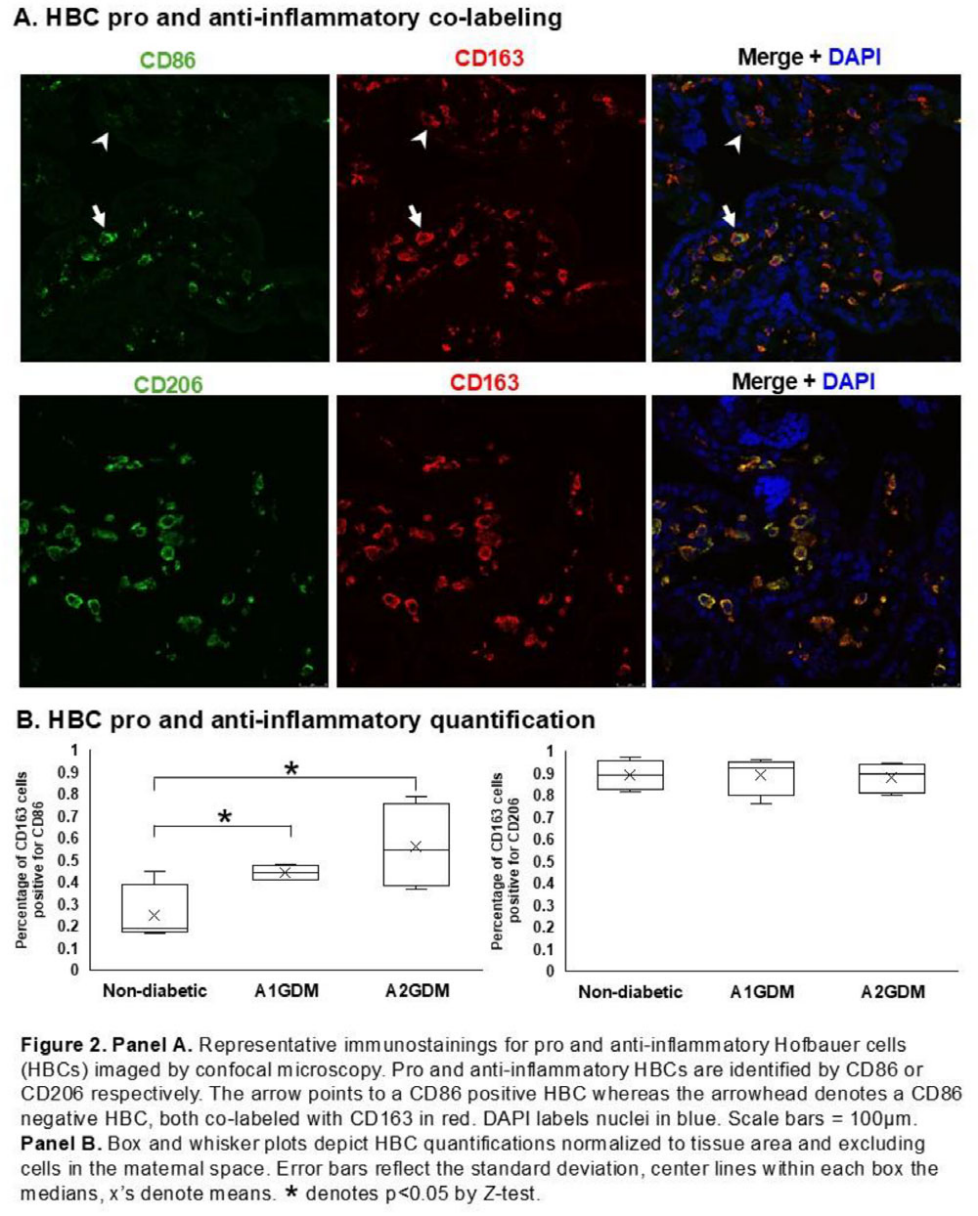

**2:00 PM – 2:15 PM**

## 23 | **Intrapartum Glucose Monitoring of A2GDM: A Randomized Control Trial**

Thomas Owens^1^; Erica Glaser^2^; Johanna A. Suskin^2^; Leonardo Antelo^2^; Guillaume Stoffels^3^; Mia Heiligenstein^4^; Xiteng Yan^2^; Zainab Al‐Ibraheemi^5^; Lois Brustman^2^

^1^Icahn School of Medicine at Mount Sinai, Atlanta, GA; ^2^Icahn School of Medicine, Mount Sinai West, New York, NY; ^3^Icahn School of Medicine at Mount Sinai, New York, NY; ^4^Icahn School of Medicine, Mount Sinai West, New York City, NY; ^5^Mount Sinai West, New York City, NY

**Objective**: The objective of this study is to assess differing intrapartum(IP) glucose monitoring protocols on initial neonatal(neo) blood glucose(BG) levels in pregnancies complicated by GDMA2

**Study Design**: This was a randomized controlled trial of singleton gestations with GDMA2 attempting a vaginal delivery. After written consent was obtained patients were randomly allocated to one of two IP glucose management protocols: the infrequent group(IN) BG measured every 4hr in latent labor, and every 2hr in active labor verses the frequent group(FR) BG measured every 2hr in latent labor, and every 1hr in active labor. Exclusion criteria included: multiple gestation, non‐compliance, poor glucose control, and/or diabetic fetopathy. The primary outcome was the first neo BG level. 74 patients were necessary to have 80% power to detect a mean difference of 10mg/dL between the two groups. Secondary outcomes included the neo BG concentrations within the first 24 hours of life, and composite neonatal adverse outcome consisting of neo hypoglycemia(HG), NICU admission, shoulder dystocia, and APGAR score at 1 minute <5.

**Results**: From 05/2024 to 5/2025 well controlled GDMA2 patients were randomized(50 in each group), and all were included in the analysis. Baseline characteristics similar between the groups, except for age, BMI and fasting GTT(Table1). The primary outcome was similar between the IF and FR group:59.0mg/dL vs 61.5 mg/dL and not statistically significant (2.5 mg/dL; 95% CI:–6.4 to 11.5; *p* = 0.57). At 24hrs of life, the mean BG level between the groups was not statistically significant,–1.0 mg/dL (95% CI:–6.4 to 4.5; *p* = 0.73). The incidence of neo HG was similar between the IF and FR groups, 26% vs 30%, (OR 1.25; 95% CI: 0.52 to 3.00, *p* = 0.63). Composite neo adverse outcome was not statistically significant between the groups (OR 1.23; 95% CI: 0.53 to 2.83; *p* = 0.63).

**Conclusion**: Our data suggests that reducing the frequency of BG monitoring in well controlled GDMA2 patients to every 4 hours in latent labor and every 2 hours in active labor, is acceptable and not associated with an increase of neonatal morbidity.

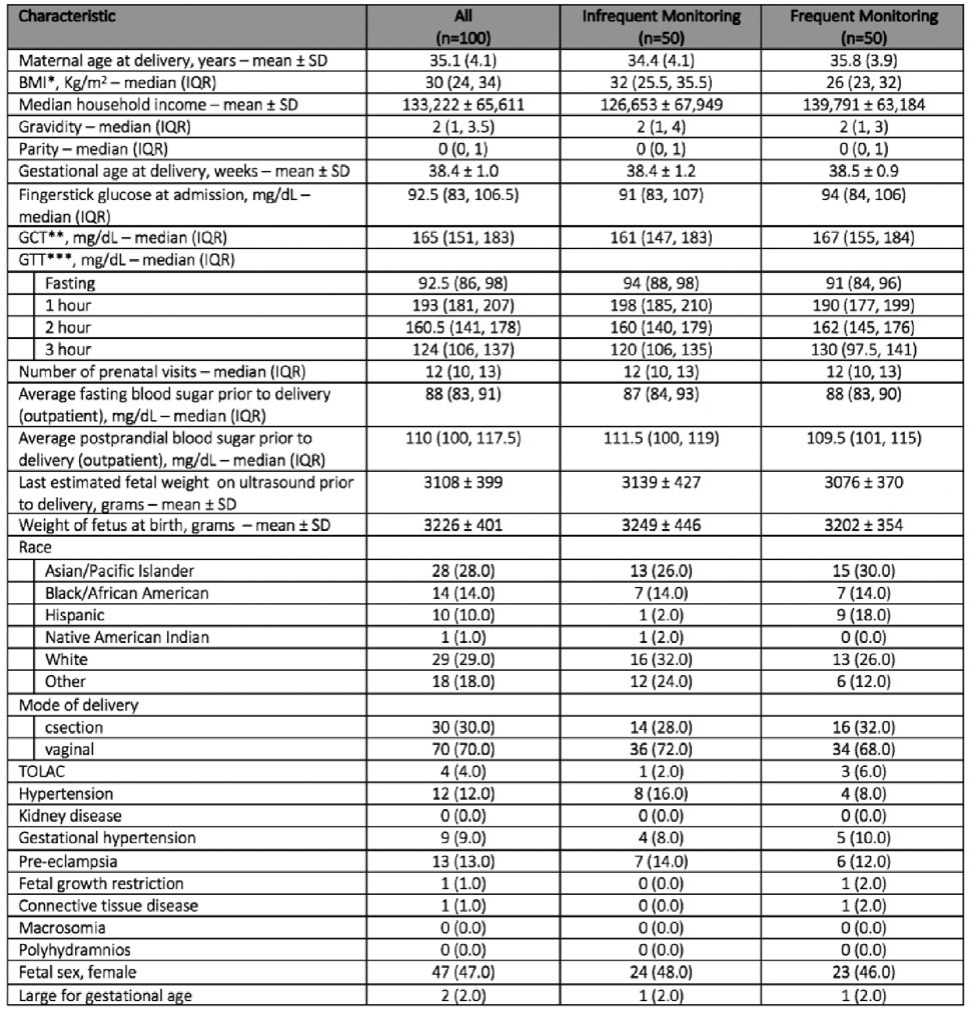

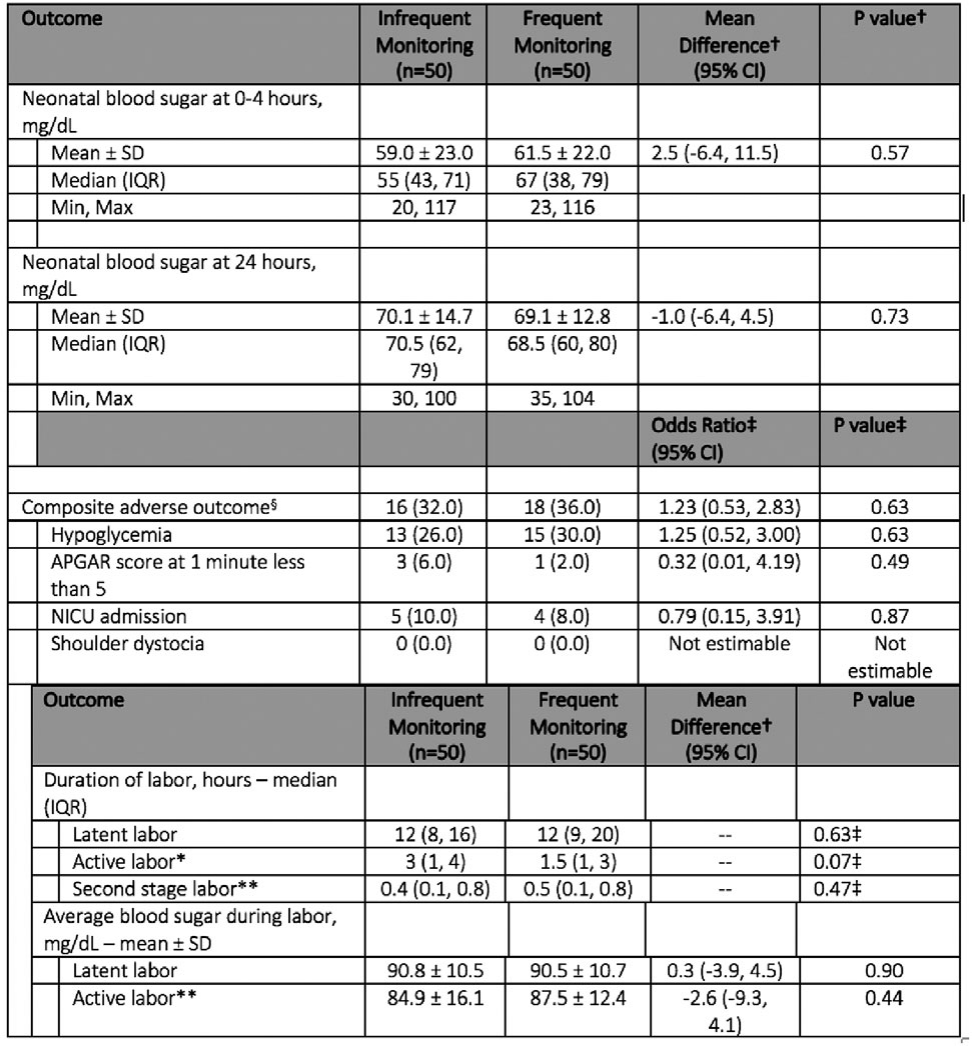

**2:15 PM – 2:30 PM**

## 24 | **Choline Supplementation and Placenta Gene Expression in GDM Pregnancies**

Youstina Soliman^1^; Isabelle Hanson^2^; Jeanette Hausser^2^; Xinyin Jiang^1^; Elisabeth A. Larson^3^; Rodney A. McLaren^1^; Itamar Futterman^4^

^1^Maimonides Medical Center, Brooklyn, NY; ^2^Department of Health and Nutrition Sciences, Brooklyn College, Brooklyn, NY; ^3^Division of Nutritional Sciences, Cornell University, Ithaca, NY; ^4^Maimonides Medical Center, Maimonides Health, NY

**Objective**: Choline supplementation improves placental angiogenesis and fetal liver health in mouse models of gestational diabetes mellitus (GDM). The objective of this planned secondary analysis was to determine the effect of maternal choline supplementation on placental gene expression in GDM‐affected pregnancies.

**Study Design**: This was a planned secondary analysis of a double‐blinded, placebo‐controlled RCT where patients with GDM were randomized to 8 weeks of either 500 mg of choline supplementation, once daily, or placebo. GDM was diagnosed by Carpenter‐Coustan criteria between 24‐ and 28‐weeks’ gestation. Full‐thickness placenta samples were collected at delivery and placental gene expression was profiled from both groups. The RNA sequencing patterns were compared between groups using DESeq2 in R studio and differentially expressed genes were identified with Benjamini‐Hocherg adjusted *p* < 0.05 and minimum detection criteria (200 normalized counts).

**Results**: Of the 45 participants recruited in this trial, nine dropped out of the study, leaving 36 (17 in the choline group vs. 19 in the placebo group) for the final analysis. There were 10 placental samples in the choline intervention and 13 samples in the placebo groups available for RNA sequencing. A total of 5609 genes were identified as differentially expressed. Two genes considered as risk factors for preeclampsia, thioredoxin (*TXN*) (fold difference: 0.6, *p* = 0.02) and peroxiredoxin 1 (*PRDX1*) (fold difference: 0.5, *p* < 0.01), were downregulated in placental samples from the choline group. Similarly, genes associated with fetal growth restriction (FGR) such as TGFB‐ induced factor homeobox 1 (*TGIF1*) (fold difference: 0.5, *p* = 0.01) and distal‐less homeobox 4 (*DLX4*) (fold difference: 0.6, *p* < 0.01) were also downregulated.

**Conclusion**: Choline supplementation in GDM pregnancies downregulated placental expression of genes associated with preeclampsia and FGR. These results suggest a potential beneficial effect of choline use in GDM pregnancies that may improve GDM birth outcomes by alleviating placental dysfunction.

**2:30 PM – 2:45 PM**

## 25 | **AI in Action: Evaluating Use of ChatGPT for Insulin Management in Gestational Diabetes Mellitus**

Lauren M. Murphy; Amanda A. Allshouse; Lauren H. Theilen; Amy Sullivan; Michelle P. Debbink; Ware Branch

University of Utah, Salt Lake City, UT

**Objective**: To compare ChatGPT management of insulin dependent gestational diabetes (A2GDM) to clinician management.

**Study Design**: In a retrospective cohort study of 60 patients with A2GDM managed with insulin and weekly glucose tracking, we compared a model using ChatGPT as a simulated clinical support tool to traditional clinician‐prescribed (CP) insulin management as recorded in the EMR. Gestational age (GA), weight, and glucose values were entered into ChatGPT with a request to create an initial insulin regimen. Subsequent log values and current doses were then entered with a request to alter the regimen as appropriate. The primary outcome was total daily insulin dose (TDD), exposure was regimen source (ChatGPT vs clinician). With paired t‐tests we compared TDD at the initial visit and at least 2 visits after insulin initiation. Difference in initial dose by trimester at initiation was analyzed with two‐sample t‐tests.

**Results**: Median GA at insulin initiation was 30 weeks. The initial CP‐prescribed TDD of insulin was 13.3 units (95%CI: 7.7‐18.9, *p* < 0.001) lower than the ChatGPT recommendation. The difference in mean TDD was significantly more pronounced among those beginning insulin in the 2nd trimester compared to the 3rd (23.7 units vs 10.1 units, *p* = 0.038). At 2–3 weeks after insulin initiation, the average CP‐prescribed increase in TDD of insulin was 10.9 units (95% CI: 7.4–14.4, *p* < 0.001) TDD prescribed at this subsequent time point was similar to ChatGPT‐recommended amount at the initial (mean [GPT‐CP]: 2.2 units (95% CI:−3. 2–7.7), *p* = 0.42), and concurrent visit (mean [GPT‐CP]: 1.6 units (−0.2–3.4), *p* = 0.08)) (Figure 1).

**Conclusion**: Clinicians initiate insulin at lower doses for A2GDM than recommended by ChatGPT. However, two weeks after insulin initiation, clinician and ChatGPT recommended doses were similar, suggesting that ChatGPT recommendations may achieve management goals more quickly. These findings suggest feasibility of prospective studies assessing AI driven A2GDM management.

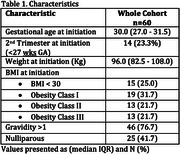

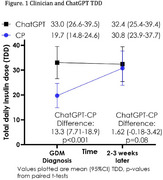

**2:45 PM – 3:00 PM**

## 26 | **Investigating Continuous Glucose Monitoring (CGM) Metrics for Gestational Diabetes to Optimize Neonatal Health**

Amy M. Valent^1^; Celeste Durnwald^2^; Katrina Ramsey^1^; Emily Fay^3^; Nicole M. Ehrhardt^3^

^1^Oregon Health & Science University, Portland, OR; ^2^Perelman School of Medicine at the University of Pennsylvania, Philadelphia, PA; ^3^University of Washington, Seattle, WA

**Objective**: Available evidence of CGM metrics shown to improve perinatal and neonatal outcomes are only among pregnancies with type 1 diabetes. Prior GDM studies have not included a sufficient sample size to identify CGM metrics linked to reduced neonatal complications. The objective is to identify CGM metrics (time in range [TIR] and mean glucose) associated with lower rates of adverse neonatal outcomes in individuals with GDM.

**Study Design**: Our study analyzed data from three RCTs using Dexcom G6 or G6 Pro CGM devices in individuals with GDM to assess the relationship between CGM metrics and composite adverse neonatal outcomes (NICU admission, transient tachypnea, respiratory distress syndrome, birth injury, or hyperbilirubinemia requiring phototherapy). Participants with <96 hours of CGM data after 24 weeks' gestation or missing outcome data were excluded. Standard pregnancy percent TIR of 63–140 mg/dl and a lower glycemic TIR 63–120 mg/dL were analyzed.

**Results**: Of 223 total participants with GDM among the 3 RCTs, 205 were included in the final analysis. Approximately 16% (*n* = 32) of the participants had a neonate with an adverse outcome. Individuals with a higher percent TIR (63–140 mg/dL) had lower neonatal composite outcome, particularly in the lower range 63‐120 mg/dL; Figure. For a one‐category difference in TIR, there was an 18% reduction in neonatal risk using the standard pregnancy TIR (63–140 mg/dL; RR 0.82 [95% CI 0.69, 0.98]) and a 21% reduction using the lower glycemic range (63–120 mg/dL; RR 0.79 [95% CI 0.64, 0.97]). Adverse neonatal outcomes were associated with higher time above range (TAR) >140mg/dL (median [IQR]: 7 [4–15] vs 4% [2–10]) and TAR >120 mg/dL (24 [14–40] vs 16% [10–27]). TIR correlated with the 24‐h mean glucose with the lowest risk among those with a 24‐h mean glucose <95 mg/dL.

**Conclusion**: Adverse neonatal outcomes are common among pregnancies complicated by GDM. For every categorical improvement in glucose TIR, there is a significant risk reduction in adverse neonatal outcomes. Future prospective studies are needed to validate these findings.

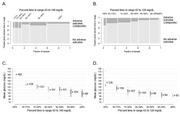

**3:00 PM – 3:15 PM**

## 27 | **Timing of Metformin Initiation for Type 2 Diabetes in Pregnancy and Perinatal Outcomes**

Claire E. Jensen^1^; Kim Boggess^2^; Kjersti M. Aagaard^3^

^1^University of North Carolina at Chapel Hill, Durham, NC; ^2^University of North Carolina at Chapel Hill, Chapel Hill, NC; ^3^HCA, Houston, TX

**Objective**: Metformin is a first‐line therapy for T2DM outside of pregnancy, which is often continued post‐conception. In pregnancy, it is an acceptable alternative to insulin, though it is unclear how timing of initiation affects fetal safety and maternal efficacy. While metformin use reduces LGA risk, we have demonstrated this occurs via loss of neonatal fat‐free mass (FFM, i.e., muscle accretion) rather than reduced adiposity. We aimed to evaluate the association between timing of metformin initiation and neonatal FFM.

**Study Design**: Secondary analysis of MOMPOD RCT of metformin 1000mg BID vs. placebo in singleton pregnancies at 11‐23 weeks with insulin‐treated early diabetes or pre‐existing T2DM. Metformin exposure was categorized as: (1) never, (2) early (preconception through randomization only), (3) continuous (preconception through delivery), and (4) late (randomization through delivery). The primary outcome was neonatal FFM, estimated from skinfold anthropometry. Secondary outcomes included composite neonatal morbidity, birthweight, SGA, LGA, maternal gestational weight gain, intrapartum IV insulin, and gestational age at delivery.

**Results**: 776 participants (never: 216, early: 175, continuous: 162, late: 223) were included. Mean gestational age for late exposure was 17.2 ± 3.7 wks. (Table 1). Adjusted analyses showed late exposure was associated with lower FFM compared to never exposure (−93.1 g, *p* = 0.045). In a planned pairwise comparison, late and continuous exposure were each associated with lower FFM compared to early exposure (late: −130 g, *p* = 0.01; continuous: −114 g, *p* = 0.03). Secondary outcomes did not differ by timing of metformin exposure, except for birthweight differences attributable to FFM in sensitivity analyses (Table 2).

**Conclusion**: Late metformin exposure was associated with lower neonatal FFM, and restricting exposure windows shows no evidence of improved maternal or neonatal outcomes.  Timing of metformin initiation appears to influence fetal lean mass development, with critical exposure windows during key periods of rapid muscle accretion in latter gestation.

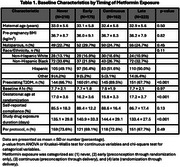

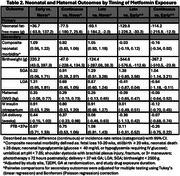

**3:15 PM – 3:30 PM**

## 28 | **Risk of Gestational Diabetes by Aerobic and Anaerobic Exercise During the First and Second Trimesters**

Takaki Tanamoto^1^; Grace Spencer^2^; Tetsuya Kawakita^3^

^1^Teine Keijinkai Hospital, Sapporo City, Hokkaido; ^2^Eastern Virginia Medical School at Old Dominion University, EVMS OBGYN, Virginia Health Sciences at Old Dominion University, VA; ^3^Eastern Virginia Medical School Department of Obstetrics and Gynecology, Macon & Joan Brock Virginia Health Sciences at Old Dominion University, Norfolk, VA

**Objective**: While some studies have shown that exercise reduces the incidence of gestational diabetes mellitus (GDM), there is limited evidence on whether aerobic or anaerobic exercise is more beneficial. This study aimed to examine the rate of GDM according to the type of exercise during early pregnancy.

**Study Design**: We analyzed data from the Nulliparous Pregnancy Outcomes Study: Monitoring Mothers‐to‐Be (nuMoM2b), a prospective cohort of individuals enrolled during the first and second trimesters. We excluded participants with pregestational diabetes or delivery <20 weeks. The primary exposure was either aerobic or anaerobic activities in the first and second trimesters (Table 1). For each trimester, the cumulative total metabolic equivalent of task (MET) was calculated for all aerobic and anaerobic activities separately. Participants were grouped as follows: “Aerobic exercise” (≧3 METs from aerobic activities), “Anaerobic exercise” (≧3 METs from anaerobic activities), “Both types of exercise” (≧3 METs in both), and “No exercise” (≦3 METs in both). Our primary outcome was GDM. Adjusted relative risks (aRR) with 95% confidence intervals (95% CI) were calculated using modified Poisson regression, adjusting for potential confounders.

**Results**: In the first and second trimesters, 9,867 and 9,264 individuals participated. Compared to those with no exercise, in the first trimester, individuals who performed aerobic exercise (aRR 0.76; 95% CI 0.61–0.96) and both types of exercise (aRR 0.59; 95% CI 0.48–0.92) had a lower risk of GDM. In the second trimester, those who performed aerobic exercise (aRR 0.68; 95% CI 0.54–0.85) and both types of exercise (aRR 0.44; 95% CI 0.26–0.76) had a lower risk of GDM (Table 2). Anaerobic exercise compared to no exercise was not associated with significant differences in GDM or other outcomes.

**Conclusion**: Aerobic exercise and combined aerobic/anaerobic exercise during the first and second trimesters were associated with a lower risk of GDM compared to no exercise. Future intervention studies focusing on exercise to prevent GDM are warranted.